# Improving Beneficial Traits in *Bacillus cabrialesii* subsp. *cabrialesii* TE3^T^ through UV-Induced Genomic Changes

**DOI:** 10.3390/plants13182578

**Published:** 2024-09-14

**Authors:** Pamela Helué Morales Sandoval, María Edith Ortega Urquieta, Valeria Valenzuela Ruíz, Kevin Montañez Acosta, Kevin Alejandro Campos Castro, Fannie I. Parra Cota, Gustavo Santoyo, Sergio de los Santos Villalobos

**Affiliations:** 1 Departamento de Ciencias Agronómicas y Veterinarias, Instituto Tecnológico de Sonora, 5 de Febrero 818 sur, Ciudad Obregón 85000, Sonora, Mexico; pamesandov37@gmail.com (P.H.M.S.); kevin.montanez219210@potros.itson.edu.mx (K.M.A.);; 2 Campo Experimental Norman E. Borlaug, Instituto Nacional de Investigaciones Forestales, Agrícolas y Pecuarias (INIFAP), Norman E. Borlaug Km. 12, Ciudad Obregón 85000, Sonora, Mexico; parra.fannie@inifap.gob.mx; 3 Instituto de Investigaciones Químico Biológicas, Universidad Michoacana de San Nicolás de Hidalgo, Morelia 58030, Michoacán, Mexico

**Keywords:** mutations, plant growth-promotion, biological control ability, swarming motility, genomics

## Abstract

It is essential to hunt for new technologies that promote sustainable practices for agroecosystems; thus, the bioprospecting of beneficial microorganisms complementing with mutation induction techniques to improve their genomic, metabolic, and functional traits is a promising strategy for the development of sustainable microbial inoculants. *Bacillus cabrialesii* subsp. *cabrialesii* strain TE3^T^, a previously recognized plant growth-promoting and biological control agent, was subjected to UV mutation induction to improve these agro-biotechnological traits. Dilutions were made which were spread on Petri dishes and placed under a 20 W UV lamp at 10-min intervals for 60 min. After the UV-induced mutation of this strain, 27 bacterial colonies showed morphological differences compared to the wild-type strain; however, only a strain named TE3^T^-UV25 showed an improvement in 53.6% of the biocontrol against *Bipolaris sorokiniana* vs. the wild-type strain, by competition of nutrient and space (only detected in the mutant strain), as well as diffusible metabolites. Furthermore, the ability to promote wheat growth was evaluated by carrying out experiments under specific greenhouse conditions, considering un-inoculated, strain TE3^T^, and strain TE3^T^-UV25 treatments. Thus, after 120 days, biometric traits in seedlings were quantified and statistical analyses were performed, which showed that strain TE3^T^-UV25 maintained its ability to promote wheat growth in comparison with the wild-type strain. On the other hand, using bioinformatics tools such as ANI, GGDC, and TYGS, the Overall Genome Relatedness Index (OGRI) and phylogenomic relationship of mutant strain TE3^T^-UV25 were performed, confirming that it changed its taxonomic affiliation from *B. cabrialesii* subsp. *cabrialesii* to *Bacillus subtilis*. In addition, genome analysis showed that the mutant, wild-type, and *B. subtilis* strains shared 3654 orthologous genes; however, a higher number of shared genes (3954) was found between the TE3^T^-UV25 mutant strain and *B. subtilis* 168, while the mutant strain shared 3703 genes with the wild-type strain. Genome mining was carried out using the AntiSMASH v7.0 web server and showed that mutant and wild-type strains shared six biosynthetic gene clusters associated with biocontrol but additionally, pulcherriminic acid cluster only was detected in the genome of the mutant strain and Rhizocticin A was exclusively detected in the genome of the wild-type strain. Finally, using the PlaBase tool, differences in the number of genes (17) associated with beneficial functions in agroecosystems were detected in the genome of the mutant vs. wild-type strain, such as biofertilization, bioremediation, colonizing plant system, competitive exclusion, phytohormone, plant immune response stimulation, putative functions, stress control, and biocontrol. Thus, the UV-induced mutation was a successful strategy to improve the bioactivity of *B. cabrialesii* subsp. *cabrialesii* TE3^T^ related to the agro-biotecnology applications. The obtained mutant strain, *B. subtilis* TE3^T^-UV25, is a promising strain to be further studied as an active ingredient for the bioformulation of bacterial inoculants to migrate sustainable agriculture.

## 1. Introduction

To meet the current food demand derived from population growth, agricultural fields have sought to increase their yields based on the use of conventional agricultural practices, such as the use of synthetic chemical fertilizers and pesticides, generating an unsustainable agroecosystem [1,2] as well as harming human health [3]. In this sense, the development of sustainable strategies to reach food security worldwide is needed. One of the most promising alternatives to conventional agricultural practices is the use of beneficial microorganisms, such as the genus *Bacillus*, which, due to their metabolic, physiologic, and ecological traits, are the most exploited genus in agricultural biotechnology [4]. Currently, *Bacillus* strains are the active ingredient in more than 85% of bacterial inoculants commercialized worldwide [5], and several species of this genus are being studied to increase crop production and yields through mechanisms such as lipopeptide production, nitrogen fixation, siderophore production, phosphate solubilization, lytic enzymes, toxins, and inducing systemic resistance in plants [6]. For this reason, bioprospecting for new strains and technologies of this genus with agro-biotechnological potential has been driven by the need to increase crop yields while minimizing the use of agrochemicals and improving soil fertility [7].

In this context, *Bacillus cabrialesii* subsp. *cabrialesii* TE3^T^, an endophytic bacteria isolated from a commercial wheat field in the Yaqui Valley located in México [8,9], can promote plant growth due to its ability to produce indoles, siderophores and solubilize phosphates [7], and has a high tolerance to thermal, hydric, and saline stress conditions [4,10]. In addition, strain TE3^T^ acts as a biological control agent (BCA) [11] against several pathogens of wheat (*Triticum turgidum* L. subsp. *durum*), such as *Bipolaris sorokiniana*, the causal agent of spot blotch, root rot, foot rot, seedling blight, and seed rot [12]. Also, it has been demonstrated that the antifungal activity of cell-free culture filtrate (CF) of strain TE3^T^ has strong antifungal activity against *B. sorokiniana* [8], reducing significantly (*p* ≤ 0.05) the visual damage, as well as the number of lesions/cm^2^ of *B. sorokiniana* vs. the control treatment [11], where the proposed metabolites involved in this biocontrol ability are surfactin, fengycin, and rhizocticin A [13]. Thus, strain TE3^T^ is a promising alternative to mitigate the negative effects of this phytopathogen on wheat, where according to climate change projections, *B. sorokiniana* is poised to emerge as a significant impediment to future wheat cultivation [14]. However, strategies to improve its beneficial role in agroecosystems need to be explored to develop more efficient and cost-effective bacterial inoculants.

Mutation induction is a viable strategy for modifying the genome of bacterial strains to improve their bioactivity [15]. The conventional random mutagenesis process is the simplest and most cost-effective technique for the improvement of bacterial capacities, either by physical and/or chemical mutagens [16]. In this sense, Ultraviolet (UV) mutagenesis is generally the most used method for improving microbes such as bacteria [17], due to the UV light being absorbed by many intracellular compounds in bacterial cells, but DNA suffers the most significant damage [18]. After DNA is exposed to ultraviolet (UV) light, photoproducts such as cyclobutane-pyrimidine dimers (CPD) and pyrimidine (6-4)-pyrimidine adducts (6-4 PP) are formed at adjacent pyrimidine sites. Dinucleotides including cytosine are major sites of UV-induced mutagenesis in bacteria and eukaryotic cells, resulting mainly in C→T transitions [18]. This damage often disrupts DNA replication, potentially leading to cell death or mutation, subsequently altering or compromising microbial traits. Examples of those mutations may manifest as shifts in colony size, cessation of sporulation, pigment loss, and a marked decrease in cell viability, known as the lethal effect [19]. However, in some cases, mutations also improve the bioactivity of bacterial stains, which can be used in agro-biotechnological applications.

At present, there are previously reported studies where, through induced random mutations, the biological potential of *Bacillus* species was improved. For example, *Bacillus* sp. ACT 1, mutated with UV irradiation and chemical methods using ethidium bromide, improved its growth kinetics and the biodegradation of Congo red [20]. Also, *Bacillus amyloliquefaciens* MF 510169 was subjected to ultraviolet (UV) light mutation to improve biodiesel production by increasing lipid accumulation. The cellular lipid content of the wild-type was improved by exposing it to UV-C (254 nm) and UV-A (365 nm) wavelengths for varying durations, ranging from 20 to 380 s [17]. Another example of improved bioactivity is *Bacillus velezensis* strain BSM54, which improved in its antifungal activity attributed to the UV mutation, as this strain produced high levels of iturin, as well as surfactin and fengycin. After UV irradiation iturin A and surfactin biosynthesis genes were expressed at higher levels in the mutant strain, and in a greenhouse assay, this mutant strain reduced more effectively (*p* ≥ 0.05) the phytopathogens *Sclerotinia sclerotiorum* (sclerotinia rot) and *Fusarium oxysporum* vs. the wild-type strain [21]. Thus, this study aimed to induce UV random mutations in *B. cabrialesii* subsp. *cabrialesii* TE3^T^ to improve traits involved in plant growth promotion and biological control against the phytopathogenic fungi *Bipolaris sorokiniana*, by seeking to increase the production of diffusible metabolites or other activities such as space and nutrient competition.

## 2. Results

### 2.1. UV-Mutation Induction of Bacillus cabrialesii subsp. cabrialesii TE3^T^, and Beneficial Traits of the Mutant Strain

**Figure 1:**
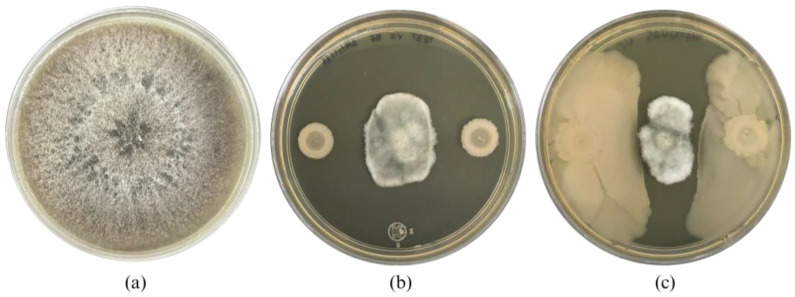
*Bipolaris sorokiniana* TPQ3 growth (**a**), and its inhibition by *Bacillus cabrialesii* subsp. *cabrialesii* TE3^T^ (**b**) and strain TE3^T^-UV25 (**c**).

After mutation induction of *Bacillus cabrialesii* subsp. *cabrialesii* TE3^T^, 27 bacterial colonies showed morphological differences compared to the wild-type strain, from the Petri dishes inoculated at a concentration of 1 × 10^−4^, 20 colonies were isolated (5 at 10 min of irradiation, 1 at 20 min, 4 at 30 min, 5 at 40 min, 1 at 50 min, and 4 at 60 min). At a concentration of 1 × 10^−5^, 4 colonies were isolated (1 at 10 min of irradiation, 2 at 20 min, and 1 at 40 min). Finally, at a concentration of 1 × 10^−6^, 3 colonies were obtained (1 at 10 min, 1 at 40 min, and 1 at 50 min). After being confronted with *B. sorokiniana* TPQ3 only one strain (TE3^T^-UV25, irradiated for 10 min at a concentration of 1 × 10^−6^) showed a significant difference related to the biological control ability compared to the wild-type strain TE3^T^, through swarming. This strain inhibited the growth of *B. sorokiniana* TPQ3 by nutrient and space competition and diffusible metabolites (4.1 ± 0.3 cm^2^ of the fungal area), which was higher compared to the type strain (6.3 ± 1.0 cm^2^), by only diffusible metabolites (Figure 1). Thus, this strain was selected for further analyses, due to its biological control ability being increased and the action mode and morphology were also different compared to the wild-type strain. These findings strongly indicate that strain TE3^T^-UV25 is a bioactivity-improved mutant strain obtained by UV irradiation.

**Figure 2:**
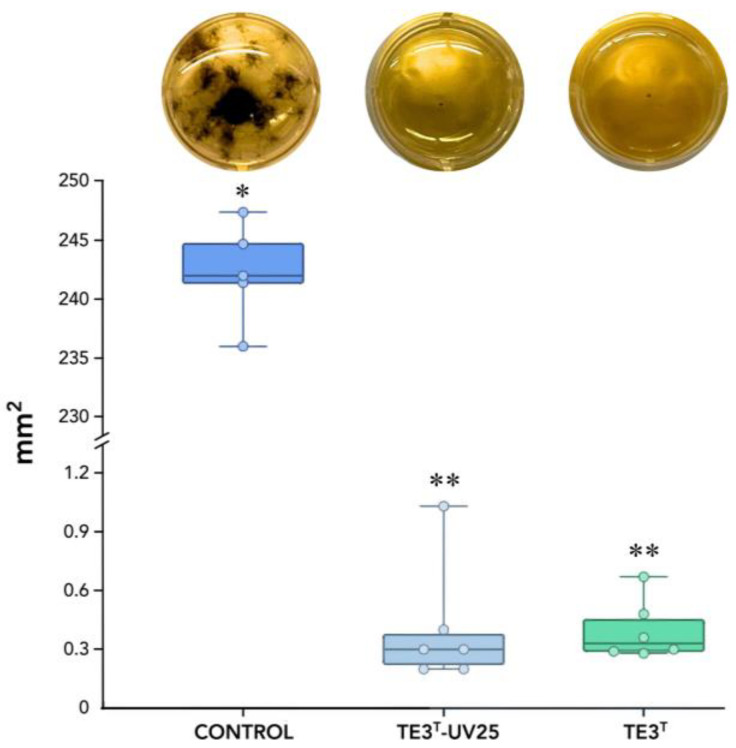
Mycelial growth of *B. sorokiniana* TPQ3, measured in mm^2^, in response to cell-free culture filtrate (CF) from the mutant TE3T-UV25 and the wild-type strain TE3^T^ grown in LB broth. * and ** Significant difference among studied strains and the control treatment (*p* ≥ 0.05). Six replicates were performed, and the *p*-value was 0.0031 according to the Kruskal–Wallis test.

The metabolites obtained after cell filtration demonstrated an inhibition rate of 99.83% for the wild-type strain TE3^T^ and 99.88% for the mutant strain (TE3^T^-UV25), with no statistically significant difference between them, in comparison with the control treatment (245.267 mm^2^), whereas the TE3^T^ strain exhibited growth of 0.396 mm^2^, and the TE3^T^-UV25 strain showed growth of 0.405 mm^2^ (Figure 2).

Although mutant strain TE3^T^-UV25 and its CF increased the biological control ability against *B. sorokiniana* compared to the wild-type strain, the plant growth-promoting effect on wheat seedlings was quantified. The results showed that no significant differences were observed in all plant biometrics between the mutant (TE3^T^-UV25) vs. type (TE3^T^) strain; however, positive significant differences were observed in comparison with the un-inoculated treatment (Table 1); for example, in shoot length (80.7%), root length (29.2%), shoot dry weight (75.0%), and root dry weight (29.3%).

### 2.2. Morphological and Biochemical Traits of Strain TE3^T^-UV25

**Figure 3:**
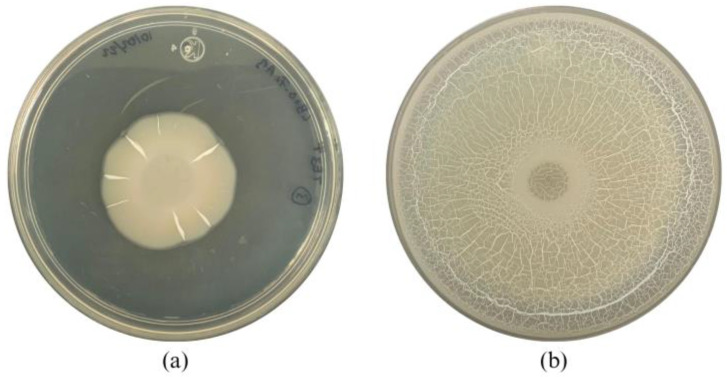
Swarming motility of *Bacillus cabrialesii* subsp. *cabrialesii* TE3^T^ (**a**) and mutant strain TE3^T^-UV25 (**b**) on LB agar.

The morphology of the wild-type strain TE3^T^ was a circular, wavy-edged colony with a high elevation and a rough, dull surface (Figure 3a and Table 2). The mutant strain TE3^T^-UV25 has an irregular colony morphology, with a wavy border, high elevation, and a glossy, milky surface (Figure 3b). In the swarming assay, the colony of the wild-type strain TE3^T^ had a growth of 2.9 ± 0.3 cm^2^, while the mutant strain TE3^T^-UV25 grew at 41.3 ± 0.8 cm^2^ (Figure 2), being 1324% higher than the wild-type strain.

On the other hand, both strains grew under the different pH levels tested. The wild-type strain TE3^T^ grows higher at pH 7, while the optimum growth of the mutant strain TE3^T^-UV25 was at pH 5. Similarly, strain TE3^T^ grew optimally up to 1% NaCl, meanwhile, strain TE3^T^-UV25 grew higher up to 2% NaCl. However, both strains tolerated the presence of 10% NaCl in the culture medium. In the hemolysis assay, strain TE3^T^ showed no change of color in the culture medium, being γ-hemolytic. However, the mutant strain presented a brown halo indicating β-hemolysis (Table 2).

To identify changes in plant growth-promoting traits, in vitro phosphate solubilization, siderophore, and indole production were assayed. The indole production for strain TE3^T^ was 3.7 ± 0.1%, while strain TE3^T^-UV25 was higher, 5.4 ± 0.3%. On the other hand, the phosphate solubilization observed by the wild-type strain was 24.4 ± 1.3%, while a reduction of 42% was observed for the mutant strain (14.1 ± 3.7%). Siderophore production was positive on both strains. For the citrate analysis, the colony of the wild-type strain changed the color medium from green to bluish, meaning that strain TE3^T^ can use citrate as a source of carbon, while strain TE3^T^-UV25 was not able to grow due to its inability to use this carbon source (Table 2).

### 2.3. Whole Genome Sequence, Hybrid Assembly, and Genomic Analyses

Illumina MiSeq platform generated a total of 1,678,639 paired-end reads (2 × 300 bp), and the MinION sequencing technology by ONT, obtained 76,145 total reads. The final hybrid assembly resulted in the closed circular chromosome of strain TE3^T^-UV25. According to the RAST annotation, the genome statistics contained 4,180,244 bp, a G + C content of 43.51%, and 4352 coding sequences (CDS) distributed into 334 subsystems, with the most significant presence of (i) amino acids and derivatives (307 CDS); (ii) carbohydrates (270); (iii) protein metabolism (212) and (iv) cofactors, vitamins, prosthetic groups, and pigments (142). Moreover, the CDS related to promoting growth activity were (i) stress response (45), such as osmotic stress (15) and oxidative stress (13); (ii) virulence, disease, and defense (36), including, resistance to antibiotics and toxic compounds (17), invasion and intracellular resistance (12) and bacteriocins, ribosomally synthesized antibacterial peptides (7); (iii) iron acquisition and metabolism (31) including siderophores (15) (Supplementary Table S1). Finally, no contamination was detected in the genome of strain TE3^T^-UV25 by CheckM.

### 2.4. Taxonomic Affiliation by Overall Genome Relatedness Index (OGRI) and Phylogenomic Relationship

Based on the full length of the 16S rRNA gene of strain TE3T-UV25, and according to the EzBioCloud database 16S-based ID, a total of 15 *Bacillus* species were identified to comply with the established similarity and completeness parameter (>98.7%) (Table 3).

Based on the Average Nucleotide Identity (ANI) cutoff value for bacterial species delimitation (≥95–96%), mutant strain TE3^T^-UV25 was taxonomically affiliated with *B. subtilis*, showing 98.29%, 98.52%, and 98.45% for ANIb, ANIm, and OrthoANI, respectively (Table 4). Similarly, the Genome-to-Genome Distant Calculator (GGDC) value (86.60%) supports the taxonomic affiliation of the mutant strain as *Bacillus subtilis,* due to the cutoff value for bacterial species delimitation is GGDC ≥ 70% (Table 4).

**Figure 4:**
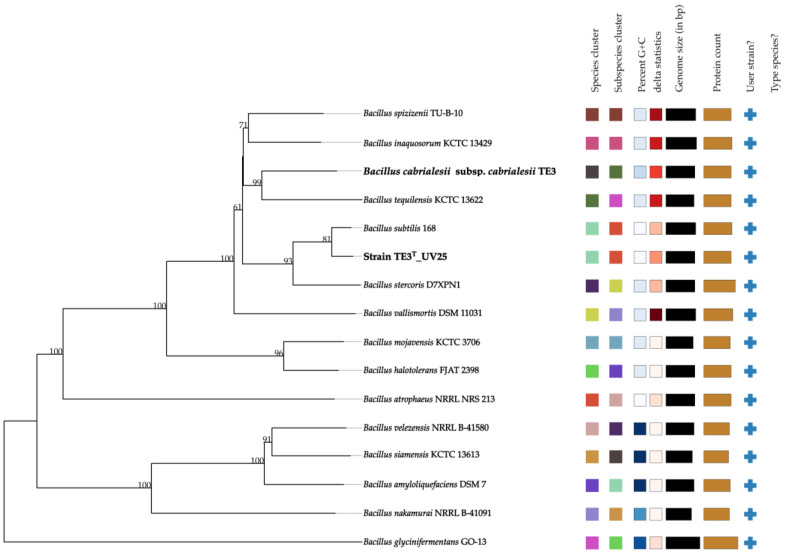
Phylogenomic relationship between mutant strain TE3^T^-UV25 and closely related species. Tree inferred with FastME 2.1.6.1 (Lefort et al., 2015 [23]) from GBDP distances calculated from genome sequences. The branch lengths are scaled in terms of the GBDP distance formula *d*5. The numbers above branches are GBDP pseudo-bootstrap support values > 60% from 100 replications, with an average branch support of 84.3%. Same color between two sequences refers that they belong to the same species and/or subspecies.

Finally, this affiliation was confirmed by creating a phylogenomic tree comparing the wild-type strain genomes available in TYGS (Figure 4). The tree demonstrates that the mutant strain TE3^T^-UV25 is clustered with *B. subtilis* (highlighted in bold), showing the same colors in both the species and subspecies clusters. This statistic evaluates phylogenomic precision based on similarity to the tree; lower values of the delta statistic indicate higher phylogenetic precision [22]. Thus, the results obtained from OGRIs (ANI and GGDC) and the phylogenomic analysis of mutant strain TE3^T^-UV25 strongly confirmed that this belongs to *Bacillus subtilis*, which indicated a large level of mutations involved in changing its taxonomic affiliation.

**Figure 5:**
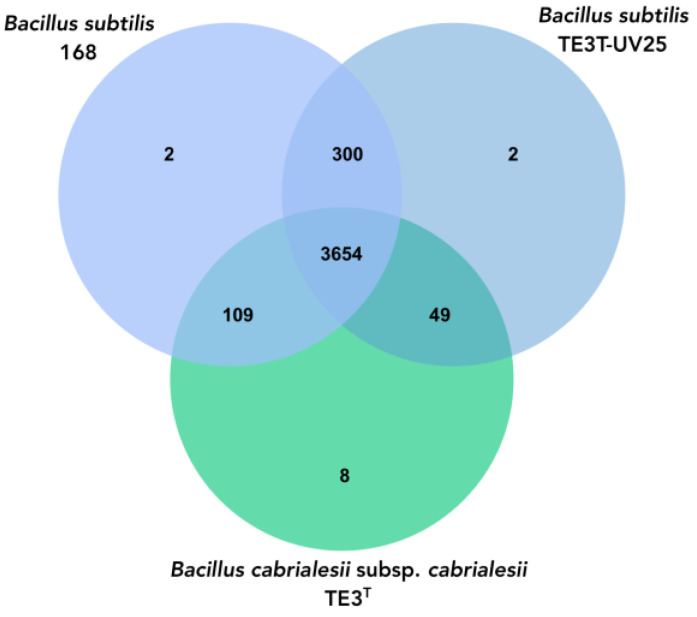
Venn diagram of the number of shared and unique genes between mutant strain TE3^T^-UV25, wild-type strain TE3^T^, and *Bacillus subtilis*, through OrthoVenn3.

To explore the phylogenomic relationship between the mutant strain, *B. cabrialesii* subsp. *cabrialesii*, and *Bacillus subtilis*, a Venn diagram was performed between these *Bacillus* species where the three bacteria share 3654 orthologous genes; however, there is a greater amount of shared genes (300) between mutant strain TE3^T^-UV25 and *B.subtilis* 168, while mutated strain shared 49 genes with the wild-type strain (Figure 5).

### 2.5. Genome Mining

The genome mining of mutant strain TE3^T^-UV25 resulted in the identification of seven biosynthesis gene clusters (BGCs), such as (i) surfactin (82%), (ii) bacillaene (100%), (iii) Fengycin (100%), (iv) bacillibactin (100%), (v) pulcherriminic acid (100%), (vi) subtilosin A (100%), and (vii) bacilysin (100%). These clusters were associated with the biosynthesis of a broad-spectrum antibacterial and antifungal activity. In contrast, genome mining of wild-type strain TE3^T^ showed seven BGCs: (i) Surfactin (86%), (ii) bacillaene (100%), (iii) Fengycin (100%), (iv) Bacillibactin (100%), (v) subtilosin A (100%), (vi) bacilysin (100%), and (vii) rhizoticin A (93%), which was not detected in the genome of strain TE3^T^-UV25, but instead the pulcherriminic acid was identified in this mutant strain (Table 5).

In the study carried out in PlaBase to observe the genes related to plant growth promotion from the PGPT-Pred analysis, 4373 genes were detected in the genome of *Bacillus cabrialesii* subsp. *cabrialesii* TE3^T^ and 4356 in mutant strain *B. subtilis* TE3^T^-UV25 (Table 6). These genes were found to be related to plant system colonization (28%), competitive exclusion (22%), stress control and biocontrol (21% for strain TE3^T^ and 20% for strain TE3^T^ -UV25), biofertilization (12%), phytohormone production (9% for strain TE3^T^ and 10% for strain TE3^T^-UV25), bioremediation (7%) and plant immune response (2%) (Table 6).

## 3. Discussion

Mutagenesis involves a permanent change in one or more nucleotides along the DNA strand at a specific site and is considered a technique to alter the genetic sequence of a bacterial strain to induce its activity [15]. Random mutagenesis by physical or chemical mutagens is a known tool to improve the capabilities of biocontrol agents and/or antifungal metabolite producers. Most mutagenic agents can damage DNA through various mechanisms, such as cleavage or deletion, addition, transversion, or base substitution. These processes can affect various pathways of the cellular machinery, generating genetic instability and, as a result, a potentially improved strain [24].

Mutation induction using UV, X-rays, γ-rays, lasers, neutrons, and chemophoresis are commonly used techniques for microorganisms [25]. UV radiation is the most essential and efficient physical method to obtain broad-spectrum mutations. Compared to chemical mutagenesis, the use of UV light as a mutagen is considered safer [26]. This bacterial mutation induction by UV irradiation ensured that different types of mutations would be generated, which could lead to improving the metabolic and ecological roles of promising wild-type strains [27,28].

The wild-type strain TE3^T^ was isolated from wheat and was reported as a promising biological control agent against *Bipolaris sorokiniana* [8,11] and plant growth promoter for wheat crops [4,29]. Then, in 2019, this strain was reported as a novel *Bacillus* species, *Bacillus cabrialesii* by de los Santos-Villalobos et al., 2019 [9]; then in 2023, this strain was the first subspecies of *B. cabrialesii*, *B. cabrialesii* subsp. *cabrialesii*, with TE3^T^ being the type strain. This strain has been studied over the past five years as a promising active component of bacterial inoculants [7]. Thus, this work aimed to improve traits involved in plant growth promotion and biological control of *Bacillus cabrialesii* subsp. *cabrialesii* TE3^T^ by UV-induced random mutations.

After 10 min of UV irradiation and incubation at 27 °C for 24 h, a bacterial colony named TE3^T^-UV25 showed significant differences in morphology compared with the wild-type strain TE3^T^. This interesting trait observed in strain TE3^T^-UV25 was the swarming motility (Figure 1 and Figure 3, and Table 2), which is associated with quick and successful growth to colonize nutrient-rich environments [30], improving its ability to control phytopathogenic fungi [31]. Thus, strain TE3^T^-UV25 showed increased inhibition of *Bipolaris sorokiniana* by 53.7% compared to the wild-type strain by both space and nutrient competition and diffusible metabolites (Figure 1 and Figure 2, and Table 2). However, wild-type strain TE3^T^ has been previously investigated and its biocontrol capabilities were only associated with the production of diffusible metabolites [13,32]; thus, in addition to competition by swarming, the remaining ability of strain TE3^T^-UV25 to biosynthesize diffusible metabolites was studied. In this sense, the cell-free supernatant of strain TE3^T^-UV25 after 72 h showed a growth inhibition of 99.9% against *B. sorokiniana*, indicating that this strain can inhibit the fungal growth by two mechanisms, (i) competition by nutrient and space, and (ii) biosynthesis of diffusible metabolites. This ability has been reported by Villa-Rodríguez (2021) where it was demonstrated that the lipopeptide complex of *Bacillus cabrialesii* subsp. *cabrialesii* TE3^T^ inhibited the growth of *Bipolaris sorokiniana* by 89.77% [11]. Both mutant and wild-type strains continue to be positive for siderophore production.

On the other hand, the mutant strain TE3^T^-UV25 not only increased its biological control capacity but also maintained its growth-promoting properties in wheat. The results show that there are no significant differences in biometric parameters between the mutant (TE3^T^-UV25) and the wild-type strain (TE3^T^). However, significant differences were observed compared to the non-inoculated treatment (Table 2), mainly highlighting stem length (44.7%), root length (22.6%), stem dry weight (42.9%) and root dry weight (22.6%). This increase in wheat biomass production by mutant strain TE3^T^-UV25 is similar to values observed for the wild-type strain TE3^T^ as is shown in this work, as well as those demonstrated in earlier experiments by Robles Montoya et al. (2019) [33]. In this analysis, the results demonstrate the ability of the mutant strain to stimulate wheat growth by increasing plant nutrient availability, as well as the production of plant growth regulators, such as IAA and indoles [34]. The genus *Bacillus* is one of the genera with the highest biocontrol and growth promotion capacity [35], being recognized as one of the most abundant and universally present in the soil, with high growth promotion attributes [36], which is consistent with the traits of the obtained mutant strain. However, there is currently little information regarding UV mutagenesis-induced PGPB to improve its ability to combat phytopathogens and promote plant growth [27]. Despite this, UV mutation is categorized as an important technique to improve functional traits in bacterial strains [37], but there is a lack of regulations for its use in the industry [38].

The metabolic characterization of mutant strain TE3^T^-UV25 showed that the optimal pH, tolerance to NaCl, and the use of citrate as a carbon source were different vs. wild-type strains. On the other hand, *B. cabrialesii* subps. *cabrialesii* TE3^T^ is a non-hemolytic strain (γ-type); however, strain TE3^T^-UV25 exhibited erythrocyte breakage (β-hemolysis). The ability to solubilize phosphates is of great relevance to their function as plant growth promoters because they play an essential role in the phosphorus (P) cycle and increase P uptake in rhizosphere soils [28]. Most phosphate solubilizing bacteria (PSB) produce indole-3-acetic acid (IAA), which enables plant cell growth and RNA/protein synthesis and increases plant growth [39]. According to Valenzuela-Aragon (2019), *B. cabrialesii* subsp. *cabrialesii* TE3^T^ can produce indoles (1.4 ± 0.1 ppm) and solubilize phosphate (43.2 ± 1.7%). In this study, the mutant strain maintained the metabolic traits of producing 5.4 ± 0.1 ppm of indoles; however, the capacity to solubilize phosphates (24.4 ± 1.3%) was significantly reduced but not missed [4]. Within the genome of the wild-type strain TE3^T^, 14 CDSs related to phosphate solubilization can be found, in contrast to the mutated strain in which only 11 of these CDSs were detected. The three missing CDSs are associated with the enzyme phosphoenolpyruvate mutase (EC 5.4.2.9) and phosphoenolpyruvate decarboxylase (EC 4.1.1.82), which are key enzymes in the catalysis of C-P bond formation by the intramolecular rearrangement of phosphoenolpyruvate to phosphoenolpyruvate (PnPy) [40]. However, despite the mutation affecting these three CDSs, plant growth promotion was not significantly impacted, indicating that this characteristic may not be entirely responsible for the observed trait.

Regarding the genomic analyses, the genome of mutant strain TE3^T^-UV25 is 54,478 bp larger compared to the genome of the wild-type strain TE3^T^ (from 1,824,296 bp to 1,818,624 bp), as well as a decrease in the G + C content of 0.69% was observed. These alterations in the genome of mutant strain TE3^T^-UV25 implied not only changes at morphological, metabolically, and ecological levels, as mentioned before, but also changes in the OGRIs values (Table 4), indicating that its taxonomic affiliation changed from *B. cabrialesii* subsp. *cabrialesii* to *B. subtilis*. Although studies on the improvement of microbial bioactivities through mutation induction have been reported, no research was found that reports that the magnitude of the mutations generated would cause a change of species, as is this case. Then as it was confirmed by the OrthoVenn diagram, in which through the comparative genomics of the platform orthologous groups were identified and annotated, inferring evolution and phylogenetic relationships between the type strain and its mutant and *Bacillus subtilis*, to which it was affiliated through the OGRIs and TYGS. Orthologous genes diverge by evolutionary speciation. Thus, orthologous genes are those shared by two or more bacteria that have an equivalent biological function and tend to be more conserved [41]. The three species shared 3564 orthologous genes and between *Bacillus cabrialesii* subsp. *cabrialesii* TE3^T^ and its mutant TE3^T^-UV25, only share 49 genes, unlike the species with which it was affiliated, *Bacillus subtilis*, which shares 300 genes, confirming the taxonomic change and affiliation.

In addition, the genome mining of mutant strain TE3^T^-UV25 revealed seven BGCs encoding secondary metabolites that confer strong antimicrobial properties (Table 5). For example, (i) Surfactin, a biosurfactant produced by large multifunctional non-ribosomal peptide synthetases (NRPSs), is crucial in combating bacterial plant diseases and inhibiting filamentous fungi. It also shows efficacy against diverse multidrug-resistant bacteria. This metabolite plays roles in cell adhesion, biofilm formation, and hemolytic activity [42,43,44,45]. Furthermore, surfactin is essential for swarming motility by reducing surface tension and acting as a wetting agent [46]; (ii) Bacillaene, a linear polyketide/non-ribosomal peptide, which enhanced the biocontrol efficiency of *Bacillus* strains, and have the ability to inhibit the growth of bacteria and showing strong antifungal activity [47]; (iii) Fengycin, a cyclic lipopeptide synthesized by NRPSs that exhibit antibacterial and antifungal activity against Gram-positive bacteria and a broad-spectrum of filamentous fungi, respectively [48,49], and also it can be involved in the development of induced systemic resistance [50]; (iv) Bacillibactin, a non-ribosomal peptide produced by NRPSs, is a catechol siderophore that binds iron with high affinity, and allows cells to obtain small amounts of iron from the environment, moreover it has a potential antibacterial and antifungal activity against plant pathogens [44,51,52]; (v) Subtilosin A, a cyclic peptide with antimicrobial activity against a wide range of bacteria, both Gram-positive, Gram-negative, aerobes and anaerobes [53,54]; (vi) Bacilysin, a non-ribosomally synthesized dipeptide antibiotic [55] that causes cell lysis in bacteria and fungi, and has also been related with the inhibition of algae [56]; and (vii) Pulcherriminic acid, which was only detected in the genome of mutant strain TE3^T^-UV25. This cyclodipeptide is one of the siderophores produced by *B. subtilis*, that acts as an iron-chelating molecule and when ligated with Fe (III) forms pulcherrimin [57]. The pulcherriminic acid can modulate iron availability and decrease oxidative stress, increasing survival and contributing to biofilm formation, while inter-species competition. In addition, it has been reported that pulcherriminic acid has an important role in the biocontrol of pathogens, this is related to iron competition [58], causing other microorganisms unable to acquire the required iron for growth acting as a protective agent [59]. Finally, Rhizocticin A was detected only in the genome of the wild-type strain, which has been reported with antifungal activity [60]; however, in metabolomics analysis realized previously in *Bacillus cabrialesii* subsp. *cabrialesii* TE3^T^, this metabolite was not identified under-tested culture conditions (28 °C and 180 rpm) and therefore it is not involved in the antifungal activity exhibited against the phytopathogen *Bipolaris sorokiniana* TPQ3 [11].

## 4. Materials and Methods

### 4.1. UV-Mutation Induction of Bacillus cabrialesii subsp. cabrialesii Strain TE3^T^

Strain TE3^T^ was obtained from the national microbial culture collection, Colección de Microorganismos Edáficos y Endófitos Nativos (COLMENA; www.itson.mx/colmena, accessed on 1 July 2021) [8,10]. After cryo-preservation (−80 °C), the bacterial cells were streaked in Petri dishes containing Luria Bertani (LB) agar as culture medium, and incubated at 27 °C for 24 h, to validate the axenicity and their morphological and biochemical traits [4]. In addition, the 16S rRNA was sequenced to confirm the strain under study [9].

Once the identity and purity of *Bacillus cabrialesii* subsp. *cabrialesii* strain TE3^T^ was confirmed, 100 µL (1 × 10^7^ cells mL^−1^) was inoculated into Eppendorf tubes containing 15 mL of LB broth and incubated at 27 °C and 180 rpm. After 24 h, the Colony Forming Units per milliliter (CFU mL^−1^) were determined, and the bacterial suspension was diluted up to 1 × 10^−6^. Later, 100 μL of each dilution were spread by triplicate into Petri dishes containing LB agar and placed at 20 cm from a 20 W UV lamp for 0, 10, 20, 30, 40, 50, and 60 min, and then these Petri dishes were incubated at 27 °C for 24 h. At this point, the colonies that showed morphological differences compared to the non-irradiated strain were further studied.

### 4.2. Plant Growth Promotion and Biological Control Ability of the Mutant Bacterial Strain

The biological control capacity of the studied strains (including strain TE3^T^ as a control treatment) was carried out using the dual confrontation technique. Thus, in the center of Petri dishes containing Potato Dextrose Agar (PDA) as a culture medium, 20 µL of *Bipolaris sorokiniana* TPQ^3^ (1 × 10^4^ conidia mL^−1^) was inoculated; then, 20 µL (1 × 10^7^ CFU mL^−1^) of each studied bacterial strains were inoculated at two equidistant points from the inoculation of the phytopathogenic fungus. After 5 days of incubation at 28 °C, the capacity of biological control was quantified (Villa-Rodriguez, 2019). This assay was carried out using five independent biological replicates and statistical analyses were performed using the STATGRAPHICS Plus ver. 5.1. Data were analyzed by one-way ANOVA and Tukey (HSD) multiple comparisons test (*p* ≤ 0.05). The bacterial strain showing an increased biological control compared with the type strain (TE3^T^) was named TE3^T^-UV25 (10 min under UV irradiation) and further studied.

To analyze the antifungal activity of extracellular metabolites from strain TE3^T^-UV25, both the wild-type and mutant strains were each inoculated at a concentration of 1 × 10^6^ CFU/mL into 20 mL of LB Broth, and incubated at 30 °C with shaking at 120 rpm. After 72 h, the cultures were centrifuged at 10,000 rpm for 25 min. The supernatant was then recovered and filtered through a hydrophilic membrane filter with a pore size of 0.22 µm. The cell-free extract (CF) was tested against *Bipolaris sorokiniana* TPQ3 (1 × 10^4^ conidia/mL) in a 24-well plate by three independent replicates. Each well contained 750 µL of LB broth, 750 µL of CF from each studied strain, and 100 µL of the phytopathogen. For the control treatment, the CF medium was replaced with sterile water. The plates were sealed and incubated with shaking at 120 rpm at 30 °C for 72 h [11]. Inhibition analysis was analyzed through STATGRAPHIC and an ANOVA test, by taking photographs and analyzing mm^2^ from images of the same size 1800 × 1800 px in ImageJ v.1.54 [61].

The growth promotion capacity of the selected strain (TE3^T^-UV25) and strain TE3^T^ was evaluated on wheat (*Triticum turgidum* subsp. *durum*) CIRNO C2008 variety, under the following greenhouse conditions: 13 h of darkness at 14 °C, 2 h of light at 18 °C, 7 h of light at 25 °C, and 2 h of light at 18 °C. For this assay, the following treatments were carried out (*n* = 100 per treatment): (i) un-inoculated, (ii) strain TE3^T^, and (iii) strain TE3^T^-UV25, under a random arrangement. A seed was sown per pot containing 40 g of soil [texture: clay, pH: 7.9 ± 0.1, organic matter (%): 1.1 ± 0.2, N (kg ha^−1^): 47 ± 4.3, P (kg ha^−1^): 58 ± 3.7, K (kg ha^−1^): 2.5 ± 0.8] collected in a commercial wheat field in the Yaqui Valley, Sonora, Mexico (27°26′08.6″ N and 109°55′57.2″ W). For the inoculated treatment, the bacteria biomass was obtained by separately inoculating 100 μL (1 × 10^7^ cells mL^−1^) of each studied strain in 250 mL of LB broth, for 24 h at 37 °C and 180 rpm; then the bacterial suspension was centrifuged at 4000 rpm (4 °C) for 10 min, and the pellet was washed twice and resuspended in sterile distilled water up to 1 × 10^8^ CFU mL^−1^ [62]. Thus, the treatments were individually inoculated with 1 mL of cell suspension during sowing, and distilled water (1 mL) was applied to the un-inoculated treatment. Each pot was irrigated with 2 mL of distilled water every 5 days; and after 120 days, the seedlings were collected to quantify: shoot height (cm), root length (cm), shoot dry weight (g), root dry weight (g), leaf number [62] Statistical analyses were carried out by using the STATGRAPHICS Plus ver. 5.1. Data were analyzed by one-way ANOVA, and Tukey (HSD) multiple comparisons test (*p* ≤ 0.05).

### 4.3. Morphological and Biochemical Characterization of Strain TE3^T^-UV25

Metabolic traits of the mutant strain TE3^T^-UV25 and the type strain TE3^T^ were carried out, as follows by using five independent biological replicates. Statistical analyses were performed using the STATGRAPHICS Plus ver. 5.1. Data were analyzed by one-way ANOVA and Tukey (HSD) multiple comparisons test (*p* ≤ 0.05).

- The swarming ability of the studied strains was carried out by inoculating 20 µL (1 × 10^7^ cells mL^−1^) of each strain into Petri dishes containing LB as a culture medium supplemented with 0.7% Bacto-Agar and 0.5% India ink. The Petri dishes were incubated for 18 h at 37 °C [31]. Then, the swarming was quantified by measuring the diameter of the colony.
- The optimal pH for the studied strains’ growth was determined in sterile 24-well plates, inoculating 100 μL (1 × 10^7^ cells mL^−1^) of each strain in 1.5 mL of LB broth at different pH values from 3 to 9, and incubated at 28 °C, and 180 rpm, for 24 h; then, the CFU mL^−1^ was determined [4].
- The bacterial growth in the presence of NaCl was quantified in a sterile 24-well plate inoculating 100 μL (1 × 10^7^ cells mL^−1^) of each strain in 1.5 mL of LB broth at 0, 1, 2, 3, 4, and 5% of NaCl. The 24-well plates were incubated at 28 °C, and 180 rpm, for 24 h; finally, the CFU mL^−1^ was determined [63].
- The citrate metabolization was determined by inoculating 20 μL (1 × 10^7^ cells mL^−1^) of each strain on Petri dishes containing Simmons citrate agar (Na_3_C_6_H_5_O_7_ 2.0 g, NaCl 5.0 g, K_2_HPO_4_ 1.0 g, NH_4_H_2_PO_4_ 1.0 g, MgSO_4_ 0.2 g, C_27_H_28_Br_2_O_5_S 0.08 g and agar 15.0 g) as a culture medium, which were incubated at 28° for 3 days. A color change to deep blue indicates a citrate utilization, while a greenish color indicates a negative result for the use of citrate as a carbon source.
- The hemolysin production was detected by inoculating 20 μL (1 × 10^7^ cells mL^−1^) of the studied strains on Petri dishes containing blood agar as a culture medium and supplemented with 5% lamb sanger. After 72 h of incubation at 28°, hemolytic activity was analyzed as reported by Sarwar et al. (2018), if no change is observed it means that there was no damage to erythrocytes, being γ-hemolytic. If a clear zone is observed around the bacterial colony indicates a breakage of erythrocytes, being β-hemolytic, and α-hemolytic occurred if a color change to dark green was observed [64].
- The indole production by strain TE3^T^-UV25 and TE3^T^ was performed using a colorimetric method based on the Salkowskýs reagent (600 mL de H_2_SO_4_ 18 M, 4.5 g de FeCl_3_) as indicated by Santos-Villalobos et al. (2012) [65]. Thus, the strains were inoculated in 30 mL of NB to which 100 ppm of tryptophan previously sterilized at 15 lb was added for 20 min. They were incubated in agitation at 28 °C for 3 days and after that, samples were centrifuged at 10,000 rpm for 10 min. Then, 1 mL of the supernatant was taken in Eppendorf tubes and centrifuged again at 8000 rpm. Quantification was performed in a 2:1 ratio by placing 100 μL of the centrifuged sample supernatant with 200 μL of Salkowski reagent on an ELISA plate. Subsequently, the plate was placed at room temperature and dark for 30 min and an absorbance reading at 549 nm was performed in a spectrophotometer.
- The siderophore production was performed by inoculating 20 μL (1 × 10^7^ cells mL^−1^) of the studied strains into Petri dishes containing Chrome Azurol S (CAS)-agar compound of four sterile solutions [66]. The first solution, a Fe-CAS indicator, is prepared with 10 mL of FeCl_3_ at 1 mM dissolved in 1 mM HCl, 50 mL of CAS at 1.21 mg/mL, and 40 mL of CTAB at 1.82 mg/mL. The second solution is obtained by dissolving 0.3 g KH_2_PO_4_, 0.5 g NaCl, and 1 g NH_4_Cl in distilled water, adding 30.24 g PIPES, and adjusting the pH to 6.8, then agar and autoclaving. The third solution is prepared with 2 g glucose, 2 g mannitol, 493 mg MgSO_4_, 11 mg CaCl_2_, 1.17 mg MnSO_4_, 1.4 mg H_3_BO_3_, 0.04 mg CuSO_4_, 1.2 mg ZnSO_4_ y 1 mg Na_2_MoO_4_ in 70 mL of H_2_0. The fourth solution is a sterilized and filtered mixture of 30 mL with 10% casamino acids. All solutions are combined, adding solution 1 at the end of the process. The strains were inoculated by loop and incubated at 28 °C for 7 days. The presence of an orange-yellow halo indicates the production of siderophores [67]. The efficiency of production (*EP*) was calculated with the formula where *DH* is the halo diameter and *DC* is the colonial diameter.

$$EP=\left(\frac{DH-DC}{DC}\right)\times 100$$

- The phosphate solubilization was carried out by inoculating 20 μL (1 × 10^7^ cells mL^−1^) of the studied strains into Petri dishes containing PVK medium [68,69], as a culture medium. The composition was 10 g (NH_4_)_2_SO_4_, 4 g NaCl, 2 g MgSO_4_·7H_2_O, 4 g KCl, 0.04 g MnSO_4_·H_2_O, 0.04 g FeSO_4_·7H_2_O and 0.025 g Bromophenol-Blue). The efficiency of solubilization (ES) was calculated as mentioned before.

### 4.4. Whole Genome Sequencing, Hybrid Assembly, and Genomic Analyses

High-quality genomic DNA was extracted from a fresh culture of strain TE3^T^-UV25. For this, 100 μL (1 × 10^7^ cells mL^−1^) were individually inoculated in 150 mL of LB broth for 24 h at 32 °C, using an orbital shaker at 120 rpm, obtaining 1 × 10^6^ CFU mL^−1^). Then, the DNA extraction was carried out following a phenol-chloroform method described by Valenzuela-Aragon et al. (2019) [4], obtaining 50 µL high-quality DNA (~150 ng µL^−1^, 260/280 ratio of ~1.9, and 260/230 ratio of ~2.1). The high-quality DNA was sequenced by using the Illumina MiSeq platform (2 × 300 bp), and the Next Generation Sequencing library preparation was carried out by using the TruSeq DNA Nano Kit for Illumina^®^ Platforms, following the manufacturer’s specified protocols [70]. Therefore, once the short-read sequencing provided by Illumina was obtained (1,678,639 paired-end reads, 2 × 300 bp) and to achieve a complete genome assembly of the studied strains, the high-quality genomic DNA was sequenced by using MinION sequencing technology by Oxford Nanopore Technology (ONT) [71], obtaining 76,145 reads.

Thus, the de novo assembly was carried out by using Unicycler version 0.4.8 [72] using short reads as a base (Illumina) and long reads (MinION) for bridging, therefore an assembly graph was performed with SPAdes version 3.13.1 [73], under default parameters, using normal mode. Unicycler polishes its final assembly with Illumina reads and Pilon to reduce the rate of small base-level errors, having a total of five polishing rounds. This assembly resulted in a closed circular chromosome of 4,180,244 bp and 43.51% G + C content for strain TE3^T^-UV25. Also, PlasmidFinder 2.0 [74] was used to identify plasmids; however, no plasmids were found. The quality of these assemblies was analyzed in the KBase online platform [75], by Quast v.4.4 [71] and CheckM v.1.0.18 [76]. As a result, no contamination was found. Finally, the genome annotation was carried out by the Rapid Annotation Using Subsystem Technology (RAST) server version 2.0 [77], using the RASTtk pipeline based on The PathoSystems Resource Integration Center (PATRIC), under default parameters [77].

### 4.5. Taxonomic Affiliation by Overall Genome Relatedness Index (OGRI) and Phylogenomic Relationship

The taxonomic affiliation of the studied strain was carried out by using the “ContEst16S” algorithm available at the EzBioCloud platform (www.ezbiocloud.net/tools/contest16s, accessed on 11 September 2024) [78], which extracted the 16S rRNA gene of the query genome and screened to determine the presence of contamination. Subsequently, the 16S rRNA gene was submitted to the 16S-based ID database at the EzBioCloud platform, in which strains showing a similarity and completeness ≥ 98.7% (threshold value) were considered for the following analyses [79]. Then, the genome sequences of those strains were used for carrying out the Overall Genome Relatedness Index (OGRI), such as The Average Nucleotide Identity (ANI) and Genome to Genome Distance Calculator (GGDC), to define species boundaries [80].

ANI was used to determine the degree of similarity between the genome of strain TE3^T^-UV25, and the more related genomes based on the cutoff value mentioned before (similarity and completeness value ≥ 98.7%). The aforementioned was carried out using the algorithm BLAST+ (ANIb) and MUMmer (ANIm) by JSpeciesWS online service [81,82]. Additionally, OrthoANI, an improved ANI algorithm based on BLASTN, available on the EzBioCloud platform was used [78]. For both analyses, the designated cutoff value for species identification is ≥95–96% [62]. Afterward, Genome to Genome Distance Calculator (GGDC) was carried out by using an online tool for the calculation of digital DNA–DNA hybridization values. GGDC. This analysis determines intergenomic sequence distances between different genomes, considering the cut-off value ≥ 70% for the delimitation of species [83]. For this, only formula 2 was considered since its calculations are independent of genome length and it is recommended for draft genomes [84].

Finally, the evolutionary relationships between strain TE3^T^-UV25, strain TE3^T^, and closely related species were constructed by the Type (Strain) Genome Server (TYGS). This web server uses GGDC (based on the established dDDH thresholds) inferred genome-scale phylogenies and cutting-edge estimates for species boundaries [22]. To compare the genome of the mutant strain with its type strain *Bacillus cabrialesii* subsp. *cabrialesii* TE3^T^ a protein sequence analysis was performed to identify orthologous groups and infer the phylogenetic relationship in these species through the OrthoVenn3 platform [85].

### 4.6. Genome Mining

The prediction of the biosynthesis pathways of secondary metabolites begins with the identification of conserved biosynthetic genes. Therefore, the genome mining of strain TE3^T^-UV25 was carried out by submitting its whole genome sequence to the AntiSMASH v7.0 web server under the “relaxed” parameter, which detected the promising biosynthetic gene clusters (BGC) associated with biocontrol [86]. This is generated by a probabilistic model to identify conserved core enzymes of secondary metabolite biosynthesis pathways [87].

Furthermore, the protein sequence FASTA file, annotated through RAST, was submitted to PGPT-Pred within PlaBAse (https://plabase.cs.uni-tuebingen.de/pb/plabase.php, accessed on 11 September 2024), a predictive resource specialized in identifying bacterial traits that promote plant growth [88].

## 5. Conclusions

Mutation induction through the use of UV radiation on *Bacillus cabrialesii* subsp. *cabrialesii* TE3^T^ was found effective, resulting in the obtaining of a mutant strain now designated as *B. subtilis* TE3^T^-UV25, which maintains the plant growth bioactivities and also has an improvement in its capacity as a biological control agent against *B. sorokiniana* TPQ^3^ compared to the wild-type strain through the space and nutrient competition (swarming motility), as well as diffusible metabolites. Therefore, it is essential to carry out genomic and metabolomic studies aimed at in-depth inspection of the genes affected and added by these mutations and their implications, such as annotation, and analysis of single nucleotide polymorphism throughout the genome, as well as the identification and quantification of the secondary metabolites produced by strain TE3^T^-UV25 and their importance.

## Figures and Tables

**Table 1:** Wheat growth promotion by mutant TE3^T^-UV25 strain and type strain (TE3T).

Parameters/Treatments	Control	TE3^T^	TE3^T^-UV25	%
Shoot length (cm)	10.9 ± 1.3 ^a^	18.8 ± 1.5 ^b^	19.7 ± 1.6 ^b^	80.7%
Root length (cm)	4.8 ± 0.6 ^a^	6.4 ± 0.5 ^b^	6.2 ± 0.7 ^b^	29.2%
Shoot dry weight (mg)	1.2 ± 0.3 ^a^	1.9 ± 0.3 ^b^	2.1 ± 0.4 ^b^	75.0%
Root dry weight (mg)	12.3 ± 2.1 ^a^	15.1 ± 2.0 ^b^	15.9 ± 1.9 ^b^	29.3%

The data presented are results and standard errors from 100 replicates. Results with the same letters are not significantly different, according to the Tukey–Kramer test (*p* ≥ 0.05).

**Table 2:** Morphological and biochemical characterization of strain TE3^T^-UV25 compared to the wild-type *Bacillus cabrialesii* subsp. *cabrialesii* TE3^T^.

	Strain TE3^T^	Strain TE3^T^-UV25
Cell morphology	Rod-shaped cells, occurring singly	Rod-shaped cells, occurring singly
Colony morphology	Wavy, raised, wough, opaque	Irregular, wavy, raised, glistening and milky
Swarming motility	2.9 ± 0.3 ^a^ cm^2^	41.3 ± 0.8 ^b^ cm^2^
pH range for growth	7	5
Growth in the presence of NaCl	1	2
Indole production	3.7 ± 0.1 ^a^ %	5.4 ± 0.3 ^b^ %
Siderophore production	Positive	Positive
Phosphate solubilization	24.4 ± 1.3 ^a^ %	14.1 ± 3.7 ^b^ %
Biocontrol activity (area of *Bipolaris sorokiniana* TPQ3)	4.1 ± 0.3 ^a^ cm^2^	6.3 ± 1.0 ^b^ cm^2^
Hemolysis	γ-hemolytic	β-hemolytic
Citrate metabolization	Positive	Negative

The statistical differences presented in the table were determined using an ANOVA test. Matching letters (“a”) indicate that the mutated strain does not differ significantly from its parental strain TE3^T^ (*p* ≥ 0.05). Conversely, differing letters (“a” and “b”) denote characteristics that exhibited significant statistical differences following the mutation.

**Table 3:** Similarity percent of the 16S rRNA gene among mutant strain TE3^T^-UV25 and closely related *Bacillus* species obtained from the EzBioCloud database.

Taxon Name	Strain Name	Accession	Similarity	Completeness
*Bacillus tequilensis*	KCTC 13622	AYTO01000043	100.00	100.00%
*Bacillus cabrialesii*	TE3T	MK462260	99.86	100.00%
*Bacillus inaquosorum*	KCTC 13429	AMXN01000021	99.86	100.00%
*Bacillus stercoris*	JCM 30051	MN536904	99.86	100.00%
*Bacillis spizizenii*	NRRL B-23049	CP002905	99.86	100.00%
*Bacillus subtilis*	NCIB 3610	ABQL01000001	99.80	100.00%
*Bacillus haloterans*	ATCC 25096	LPVF01000003	99.73	100.00%
*Bacillus vallismortis*	DV1-F-3	JH600273	99.66	100.00%
*Bacillus velezensis*	CR-502	AY603658	99.64	100.00%
*Baciilus mojavensis*	RO-H-1	JH600280	99.52	100.00%
*Bacillus nakamurai*	NRRL B-41091	LSAZ01000028	99.52	100.00%
*Bacillus siamensis*	KCTC 13613	AJVF01000043	99.39	100.00%
*Bacillus amyloliquefaciens*	DSM 7	FN597644	99.25	100.00%
*Bacillus atrophaeus*	JCM 9070	AB021181	99.18	100.00%
*Bacillus glycinifermantans*	GO-13	LECW01000063	98.70	100.00%

**Table 4:** Average nucleotide identity (ANI) and Genome to Genome Distance Calculator (GGDC) values of mutant strain TE3^T^-UV25 and closely related *Bacillus* species.

Taxon Name	Strain	Accession Number	ANIb	ANIm	Ortho ANI Value (%)	GGDC Formula 2
*Bacillus tequilensis*	KCTC 13622(T)	GCA_000507145.1	91.35	91.82	91.69	44.90
*B. cabrialesii* subsp. *cabrialesii*	TE3(T)	GCA_004124315.1	92.24	92.49	92.44	47.80
*B. inaquosorum*	KCTC 13429(T)	GCA_003148415.1	92.83	93.10	93.00	50.30
***B. subtillis***	**168**	**GCA_000009045.1**	**98.29**	**98.52**	**98.45**	**86.60**
*B. stercoris*	D7XPN1(T)	GCA_000738015.1	95.09	95.33	95.31	61.90
*B. spizizenii*	TU-B-10(T)	GCA_000227465.1	92.86	93.22	93.18	50.90
*B. velezensis*	NRRL B-41580(T)	GCA_001461825.1	76.44	84.17	76.97	20.60
*B.halotolerans*	FJAT-2398(T)	GCA_001637525.1	87.09	87.78	87.52	32.90
*B. mojavensis*	RO-H-1	GCA_000245335.1	86.95	87.56	87.21	32.50
*B. vallismortis*	DSM 11031(T)	GCA_004116955.1	90.66	91.23	91.12	42.90
*B. nakamurai*	NRRL B-41091(T)	GCA_001584325.1	76.83	83.54	77.35	20.60
*B. siamensis*	KCTC 13613(T)	GCA_000262045.1	76.50	84.43	77.31	20.80
*B. amyloliquefaciens*	DSM 7(T)	GCA_000196735.1	76.35	84.54	77.13	20.90
*B. atrophaeus*	NRRL NRS 213(T)	GCA_001584335.1	79.32	83.91	79.50	22.20
*B. glycinifermentans*	GO-13(T)	GCA_001042475.2	72.51	85.42	72.92	19.30

**Table 5:** BGCs identified in the genome of mutant strain TE3^T^-UV25 and wild-type strain TE3^T^, according to AntiSMASH.

BCG	Type	TE3^T^-UV25	TE3^T^
Bacillaene ^1^	PKS	100%	100%
Fengycin ^1^	NRPS	100%	100%
Bacillibactin ^1^	NRPS	100%	100%
Subtilosin A ^2^	RiPP; Thiopeptide	100%	100%
Bacilysin ^1^	Other	100%	100%
Surfactin ^1^	NRPS	82%	86%
Rhizocticin A ^3^	Other	0%	93%
Pulcherriminic acid ^1^	Other	100%	0%

The numbers above the BGC name correspond to ^1^ Antibacterial and antifungal, ^2^ Antibacterial and ^3^ Antifungal activity.

**Table 6:** Frequency of genes involved in features of plant growth-promoting traits in the studied strains, annotated in PlaBase.

Features	Strain TE3^T^	Strain TE3^T^-UV25
Biofertilization	503	509
Bioremediation	286	289
Colonizing plant system	1225	1198
Competitive exclusion	955	961
Phytohormone	417	406
Plant immune response stimulation	91	88
Putative functions	9	9
Stress control and biocontrol	887	896
**Total of genes**	**4373**	**4356**

## Data Availability

The draft genome sequence has been deposited in DDBJ/ENA/GenBank under accession number 0000. The version described in this paper is the first version, 0000, under BioProject number PRJNA1138311 and BioSample number SAMN42691756. The raw data files may be consulted under the following accession number SRX25401844 and SRX25401843, under the following link https://www.ncbi.nlm.nih.gov/sra/SRX25401843[accn], https://www.ncbi.nlm.nih.gov/sra/SRX25401844[accn], accessed on 11 September 2024.

## Supplementary Materials

The following supporting information can be downloaded at: https://www.mdpi.com/article/10.3390/plants13182578/s1, Table S1: Features of strain TE3^T^-UV25 genome by RAST (response to osmotic stress, oxidative stress, resistance to antibiotics and toxic compounds, invasion and intracellular resistance, bacteriocins, ribosomally synthesized antibacterial peptides, iron acquisition and metabolism, phosphorus metabolism and auxin biosynthesis).
