# Supplementary material for: Improving Beneficial Traits in Bacillus cabrialesii subsp. cabrialesii TE3T through UV-Induced Genomic Changes
Source: Plants (Basel). 2024 Sep 14;13(18):2578. doi: 10.3390/plants13182578 (PMC11434716; doi:10.3390/plants13182578)
Supplement: Supplementary file 1 [file plants-13-02578-s001.zip › plants-3166571-supplementary.pdf]

Improving Beneficial Traits in Bacillus cabrialesii subsp. cabrialesii TE3T through UV-Induced Genomic Changes

Pamela Helué Morales Sandoval 1,†, María Edith Ortega Urquieta 1,†, Valeria Valenzuela Ruíz 1, Kevin Montañez Acosta 1, Kevin Alejandro Campos Castro 1, Fannie I. Parra Cota 2, Gustavo Santoyo 3 and Sergio de los Santos Villalobos 1,*

[1] Departamento de Ciencias Agronómicas y Veterinarias, Instituto Tecnológico de Sonora, 5 de Febrero 818 sur, Ciudad Obregón 85000, Sonora, Mexico
[2] Campo Experimental Norman E. Borlaug, Instituto Nacional de Investigaciones Forestales, Agrícolas y Pecuarias (INIFAP), Norman E. Borlaug Km. 12, Ciudad Obregón 85000, Sonora, Mexico
[3] Instituto de Investigaciones Químico Biológicas, Universidad Michoacana de San Nicolás de Hidalgo, Morelia 58030, Michoacán, Mexico.
*Correspondance: Sergio de los Santos-Villalobos, sergio.delossantos@itson.edu.mx
†These authors contributed equally to this work.

Table S1. Features of strain TE3^{T}-UV25 genome by RAST (response to osmotic stress, oxidative stress, resistance to antibiotics and toxic compounds, invasion, intracellular resistance, bacteriocins, ribosomally synthesized antibacterial peptides, iron acquisition and metabolism, phosphorus metabolism, and auxin biosynthesis).

Protein	Function
	**Response to osmotic stress**
BetB	Betaine aldehyde dehydrogenase (EC 1.2.1.8)
OpuD	Glycine betaine transporter OpuD
OpuAA	Glycine betaine ABC transport system, ATP-binding protein OpuAA (EC 3.6.3.32)
OpuAB	Glycine betaine ABC transport system, permease protein OpuAB
OpuAC	Glycine betaine ABC transport system, glycine betaine-binding protein OpuAC
OpuC	Glycine betaine/L-proline ABC transporter, glycine betaine/L-proline- binding/permease protein
OpuBA	Choline ABC transport system, ATP-binding protein OpuBA
OpuBB	Choline ABC transport system, permease protein OpuBB
OpuBC	Choline ABC transport system, choline-binding protein OpuBC
OpuBD	Choline ABC transport system, permease protein OpuBD
GbsB	Alcohol dehydrogenase GbsB (type III), essential for the utilization of choline (EC 1.1.1.1)
Glycerol	Glycerol uptake facilitator protein
	**Response to oxidative stress**
GPX	Glutathione peroxidase (EC 1.11.1.9)
sodB	Superoxide dismutase [Fe] (EC 1.15.1.1)
sodC	Superoxide dismutase [Cu-Zn] precursor (EC 1.15.1.1)
NSTR	Nitrite-sensitive transcriptional repressor NsrR
PerR	Peroxide stress regulator
Furp	Ferric uptake regulation protein
AhpC	Alkyl hydroperoxide reductase subunit C-like protein

Osmcl	Organic hydroperoxide resistance transcriptional regulator
OsmclR	Organic hydroperoxide resistance protein
SODA	Manganese superoxide dismutase (EC 1.15.1.1)
SODB	Superoxide dismutase [Fe] (EC 1.15.1.1)
SODC	Superoxide dismutase [Cu-Zn] precursor (EC 1.15.1.1)
SOR	Superoxide reductase (EC 1.15.1.2)
SODFe/Mn	Superoxide dismutase [Mn/Fe] (EC 1.15.1.1)
SODMn	Superoxide dismutase [Mn] (EC 1.15.1.1)
SODFe/Zn	superoxide dismutase [Fe-Zn] (EC 1.15.1.1)
	**Resistance to antibiotics and toxic compounds**
CZCR	Cobalt-zinc-cadmium resistance protein
czcD	Cobalt-zinc-cadmium resistance protein CzcD
TRCd	Cd(II)/Pb(II)-responsive transcriptional regulator
TRMer	Transcriptional regulator, MerR family
HmrR	Heavy metal resistance transcriptional regulator HmrR
CIA	Copper-translocating P-type ATPase (EC 3.6.3.4)
CSA	Copper/silver efflux P-type ATPase
CopC	Copper resistance protein CopC
CopD	Copper resistance protein CopD
gyrA	DNA gyrase subunit A (EC 5.99.1.3)
gyrB	DNA gyrase subunit B (EC 5.99.1.3)
SatA	Streptothricin acetyltransferase, Streptomyces lavendulae type
BLI	Metal-dependent hydrolases of the beta-lactamase superfamily I
TetR	Transcription regulator of multidrug efflux pump operon, TetR (AcrR) family
bsh	Choloylglycine hydrolase (EC 3.5.1.24)
FosB	Fosfomycin resistance protein FosB
	**Invasion and intracellular resistance**
Rv0682	SSU ribosomal protein S12p (S23e)
Rv0683	SSU ribosomal protein S7p (S5e)
Rv0684	Translation elongation factor G
Rv0685	Translation elongation factor Tu
Rv1594	Quinolinate synthetase (EC 2.5.1.72)
Rv1595	L-aspartate oxidase (EC 1.4.3.16)
Rv1596	Quinolinate phosphoribosyltransferase [decarboxylating] (EC 2.4.2.19)
Rv0667	DNA-directed RNA polymerase beta subunit (EC 2.7.7.6)
Rv0668	DNA-directed RNA polymerase beta' subunit (EC 2.7.7.6)
Rv1641	Translation initiation factor 3
Rv1642	LSU ribosomal protein L35p
Rv1643	LSU ribosomal protein L20p
	**Bacteriocins, ribosomally synthesized antibacterial peptides**
BceB	Bacitracin export permease protein BceB
BceA	Bacitracin export ATP-binding protein BceA

BceS	Two-component sensor histidine kinase BceS
BceR	Two-component response regulator BceR
LiaG	Conserved protein LiaG in B. subtilis in Lia cluster
LiaH	Protein LiaH, similar to phage shock protein A
LiaI	Protein LiaI

Iron acquisition and metabolism

A	Sortase A, LPXTG specific
HtsA	Heme ABC type transporter HtsABC, heme-binding protein
HtsB	Heme ABC type transporter HtsABC, permease protein HtsB
HtsC	Heme ABC type transporter HtsABC, permease protein HtsC
Hyp?	Antibiotic biosynthesis monooxygenase domain-containing protein
X-ABC1	Uncharacterized iron compound ABC uptake transporter, substrate-binding protein
X-ABC2	Uncharacterized iron compound ABC uptake transporter, permease protein
X-ABC3	Uncharacterized iron compound ABC uptake transporter, ATP-binding protein
ZnH	Zn-dependent hydrolase YycJ/WalJ, required for cell wall metabolism and coordination of cell division with DNA replication
R	Two-component response regulator SA14-24
S	Two-component sensor kinase SA14-24
EfeU	Ferrous iron transport permease EfeU
EfeO	Ferrous iron transport periplasmic protein EfeO, contains peptidase-M75 domain and (frequently) cupredoxin-like domain
EfeB	Ferrous iron transport peroxidase EfeB
dhbA	2,3-dihydro-2,3-dihydroxybenzoate dehydrogenase (EC 1.3.1.28) [bacillibactin] siderophore
dhbB	Isochorismatase (EC 3.3.2.1) [bacillibactin] siderophore
dhbC	Isochorismate synthase (EC 5.4.4.2) [bacillibactin] siderophore
dhbE	2,3-dihydroxybenzoate-AMP ligase (EC 2.7.7.58) [bacillibactin] siderophore
dhbF	Bacillibactin synthetase component F (EC 2.7.7.-)
yuiI	Trilactone hydrolase [bacillibactin] siderophore
feuA	Fe-bacillibactin uptake system FeuA, Fe-bacillibactin binding
feuB	Fe-bacillibactin uptake system FeuB
feuC	Fe-bacillibactin uptake system FeuC
Hyp1	Antibiotic biosynthesis monooxygenase domain-containing protein
X-ABC1	Uncharacterized iron compound ABC uptake transporter, substrate-binding protein
X-ABC2	Uncharacterized iron compound ABC uptake transporter, permease protein
X-ABC3	Uncharacterized iron compound ABC uptake transporter, ATP-binding protein
IPP	Inorganic pyrophosphatase (EC 3.6.1.1)
PhoHv	Phosphate starvation-inducible protein PhoH, predicted ATPase
PhoH?	Predicted ATPase related to phosphate starvation-inducible protein PhoH
SAP	secreted alkaline phosphatase
AP	Alkaline phosphatase (EC 3.1.3.1)
APl	Alkaline phosphatase like protein
AP?	putative alkaline phosphatase-like protein
phoP	Alkaline phosphatase synthesis transcriptional regulatory protein PhoP
PLAT	Probable low-affinity inorganic phosphate transporter
NaPi	Sodium-dependent phosphate transporter

PhoR	Phosphate regulon sensor protein PhoR (SphS) (EC 2.7.13.3)
PpaX	Inorganic pyrophospatase PpaX (EC 3.1.3.18)

Auxin metabolism

APRT	Anthranilate phosphoribosyltransferase (EC 2.4.2.18)
PRAI	Phosphoribosylanthranilate isomerase (EC 5.3.1.24)
TSa	Tryptophan synthase alpha chain (EC 4.2.1.20)
TSb	Tryptophan synthase beta chain (EC 4.2.1.20)
